# Apoptotic Effects of *Agapanthus africanus* Extracts and Identification of Volatile Compounds from the *n*-Butanol Fraction

**DOI:** 10.3390/molecules31071062

**Published:** 2026-03-24

**Authors:** Makgwale S. Mphahlele, Kingsley C. Mbara, Daniel M. Tswaledi, Raymond T. Makola, Clemence Tarirai, Jeremia L. Shai

**Affiliations:** 1 Department of Biomedical Sciences, Tshwane University of Technology, Private Bag X680, Pretoria 0001, South Africa; shailj@tut.ac.za; 2 Nanomedicines Manufacturing, Biopharmaceutics and Diagnostics Research Laboratory, Department of Pharmaceutical Sciences, Tshwane University of Technology, Private Bag X680, Pretoria 0001, South Africa; tariraic@tut.ac.za; 3 Department of Biochemistry and Biotechnology, School of Science and Technology, Sefako Makgatho Health Sciences University, Pretoria 0204, South Africa; lefatswaledi@gmail.com; 4 Department of Biochemistry, Microbiology and Biotechnology, School of Molecular and Life Science, University of Limpopo, Turflop Campus, Sovenga 0727, South Africa; raymond.makola@ul.ac.za

**Keywords:** *Agapanthus africanus*, apoptosis, anticancer, cytotoxicity, gas chromatography-mass spectrometry, phytochemicals

## Abstract

*Agapanthus africanus* (L.) Hoffmanns. is a medicinal plant traditionally used in South Africa for its promise as a source of bioactive compounds with anticancer properties. This study aimed to investigate the apoptotic effects of *A. africanus* fractions on cancer cell lines and to identify the bioactive phytochemical constituents using gas chromatography-mass spectrometry analysis. To test for cytotoxicity, MCF-7, A549, and HeLa cancer cells were treated with crude extract, *n*-hexane, *n*-butanol, dichloromethane, and aqueous fractions of *A. africanus* extracts at different concentrations (0.00–1000 µg/mL). Total apoptosis was quantified using Annexin V/PI staining. The 4′,6-diamidino-2-phenylindole was used to detect nuclear morphological changes and the Caspase-GLO 3/7 assay was employed to check the caspase activation in the cancer cells. Expression of apoptosis-related (*caspase-3*, *bax*, *bcl-2*) genes was evaluated using real time-polymerase chain reaction. The crude extract of *A. africanus* exhibited dose-dependent cytotoxicity against MCF-7, A549, and HeLa cells, with IC_50_ values of 130 µg/mL, 380 µg/mL, and <125 µg/mL, respectively. Among the tested fractions, the *n*-butanol fraction showed cytotoxicity towards MCF-7 cells with an IC_50_ value of <870 µg/mL. In contrast, *n*-hexane, dichloromethane and the aqueous fractions exhibited higher IC_50_ values against cancer cells. Flow cytometry analysis, which was applied to quantify total apoptosis, revealed that the crude extract of *A. africanus* induced apoptosis by (~60%) compared to the *n*-butanol fraction, which exhibited a moderate apoptotic effect (~27%). DAPI nuclear staining showed nuclear shrinkage and chromatin condensation in the MCF-7 cell line, whereas in Caspase-GLO 3/7, the crude extract and *n*-butanol fraction resulted in significant luminescence, indicating activation of caspase-3/7. Caspase-3/7 analysis showed *A. africanus* treatments produced varying levels of apoptotic activation. The crude extract increased caspase activity by 2.9-fold, while the *n*-butanol fraction induced a 1.7-fold rise compared with untreated cells. GC-MS chromatograms detected and identified 16 compounds in the fractionated *n*-butanol and 23 compounds from the crude extract of *A. africanus*. The major compounds identified from the *n*-butanol fraction included *n*-hexadecanoic acid; α-tocopherol and 9,12,15-octadecatrienoic acid, while the GC–MS profile of the crude extract was dominated by 6,10,14-trimethylpentadecan-2-one; 1,3,5-Triphenylcyclohexane and phytol. The study indicates the pro-apoptotic potential of *A. africanus*, particularly in its crude form, supporting its ethnopharmacological use and suggesting its relevance as a candidate for anticancer drug discovery.

## 1. Introduction

Cancer is a significant global health issue, with approximately 19.3 million new cases and roughly 10 million fatalities documented worldwide in 2020 [1,2]. In South Africa, the incidence of cancer is escalating, as reported by the National Cancer Registry (NCR), which documented over 111,321 new cancer cases, 282,418 existing cases, and 64,547 cancer-related fatalities in 2022 [3,4]. There has been a substantial rise of almost 50% in cancer incidence from 2008 to 2019 [1,2,3,4]. Contributing factors to this increase encompass an ageing population, urbanisation, and alterations in lifestyle choices, including smoking, alcohol consumption, poor diet, and physical inactivity [2]. Current chemotherapeutic strategies, including chemotherapy, surgery, and radiotherapy, are often hampered by systemic toxicity, drug resistance, and nonspecific targeting of healthy tissues [5]. These barriers have heightened the pursuit of new, safer, and more selective anticancer agents, particularly from natural products, which remain a valuable source of bioactive molecules for drug discovery [6].

**Figure 1:**
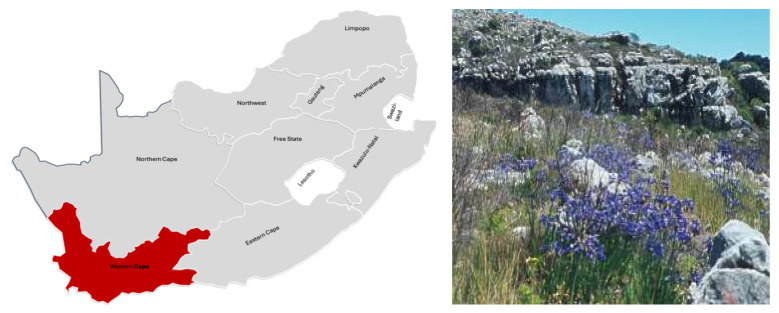
The natural distribution of *Agapanthus africanus* in South Africa, showing its restriction to the Western Cape Province within the winter-rainfall region [7].

*Agapanthus africanus* (L.) Hoffmanns., known as the African lily or lily of the Nile, is a rhizomatous perennial herb in the *Amaryllidaceae* family. It is native to South Africa, primarily distributed in the Western Cape province, thriving in the fynbos environment [7]. The species is distinguished by its evergreen, strap-shaped leaves and clusters of violet to blue flowers, enhancing its significant horticultural and ecological worth [8]. *A. africanus* occurs predominantly on the rocky slopes, coastal cliffs, and mountain terrain, favouring acidic sandy soils derived from Table Mountain sandstone [9]. Its indigenous distribution spans from the Cape Peninsula to Stellenbosch, Hermanus, and Caledon in the Western Cape (see Figure 1) [10].

*A. africanus* has long been used in traditional medicine to manage pregnancy-related ailments, coughs, heart diseases, and inflammation [11,12]. Although primarily a wild species, *A. africanus* is also cultivated ornamentally and commercially propagated through nurseries and botanical gardens, which helps reduce harvesting pressure on wild populations. Pharmacological studies support these traditional uses, reporting uterotonic activity and identifying saponins, flavonoids, and phenolic compounds as active constituents [13]. *A. africanus* has been investigated for its phytochemicals, which showed the presence of different bioactive compounds that may contribute to its cytotoxic and pro-apoptotic effects [13]. Phytochemicals such as steroidal saponins, phenolic compounds including flavonoids, alkaloids, phytosterols, coumarins, and volatile compounds have been identified [14]. These phytochemicals are characterised by their pharmacological functions, especially the ability to modulate oxidative stress, the inhibition of cell proliferation, and the induction of apoptosis [14]. For example, steroidal saponins are well known to permeabilise the cell membrane and trigger apoptosis through caspase activation and mitochondrial dysfunction, and phenolic compounds are well-studied to serve as antioxidants, especially in cancer cells, which already contain high levels of reactive oxygen species (ROS) [15]. Phenolic compounds such as quercetin and curcumin induce ROS-driven cell death in a range of cancer cell types [16]. Therefore, continued investigation into the phytochemical constituents of *A. africanus* and their mechanisms of action in cancer therapy is imperative.

Apoptosis is one of the most effective strategies for the selective elimination of cancer cells and helps maintain healthy tissue by a well-defined sequence of changes in the cells [17]. Apoptosis can occur through the intrinsic pathway (mitochondria-mediated) and the extrinsic pathway (death receptor-mediated), which converge on the activation of caspases, which drive the cell towards its controlled self-destruction [18]. Caspases-3 and -7 are executioner enzymes in the apoptotic pathway and are essential for the last phase of cell death. The intrinsic pathway is triggered by internal stressors such as DNA damage or oxidative stress. These signals lead to mitochondrial outer membrane permeability, which causes the release of pro-apoptotic factors such as cytochrome C into the cytoplasm. The intrinsic pathway is often regulated by the *bcl-2* protein family, which can be either pro-apoptotic (e.g., *bax*, *bak*) or anti-apoptotic (e.g., *bcl-2*, *Bcl-xl*) [17,18]. On the other hand, the extrinsic pathway begins with the binding of extracellular death ligands, such as tumour necrosis factor-alpha, to their corresponding cell-surface death receptors. This interaction forms the death-inducing signalling complex, which then activates caspase-8 [17].

Previous investigations, including preliminary work from our laboratory (unpublished data), have demonstrated the cytotoxic potential and suggest that volatile compounds derived from *A. africanus* possess anticancer effects in vitro. Furthermore, recent studies have reported compounds such as *agapanthussaponin*, steroidal saponins, and phenolic compounds that exhibit apoptosis-induced effects in small-cell lung cancer models [13,14,19,20]. However, there remains limited evidence on the fraction-dependent cytotoxicity, apoptotic mechanisms, and chemical composition of this species, in relation to breast, cervical, and lung cancer. Given the rising incidence of drug-resistant cancers and the need for alternative, safer chemotherapeutics, investigating the apoptotic mechanisms of the volatile compounds from *A. africanus* extracts could lead to the discovery of novel therapeutic leads for oncology with improved efficacy and safety profiles. This study evaluated the cytotoxicity and apoptotic effects of *A. africanus* extract on cancer cells and identified its volatile compounds, thereby establishing a mechanistic basis for its potential anticancer applications.

## 2. Results

### 2.1. Effect of the Cell Viability of Crude Extract and Fractionated A. africanus on MCF-7, A549, and HeLa Cells

**Figure 2:**
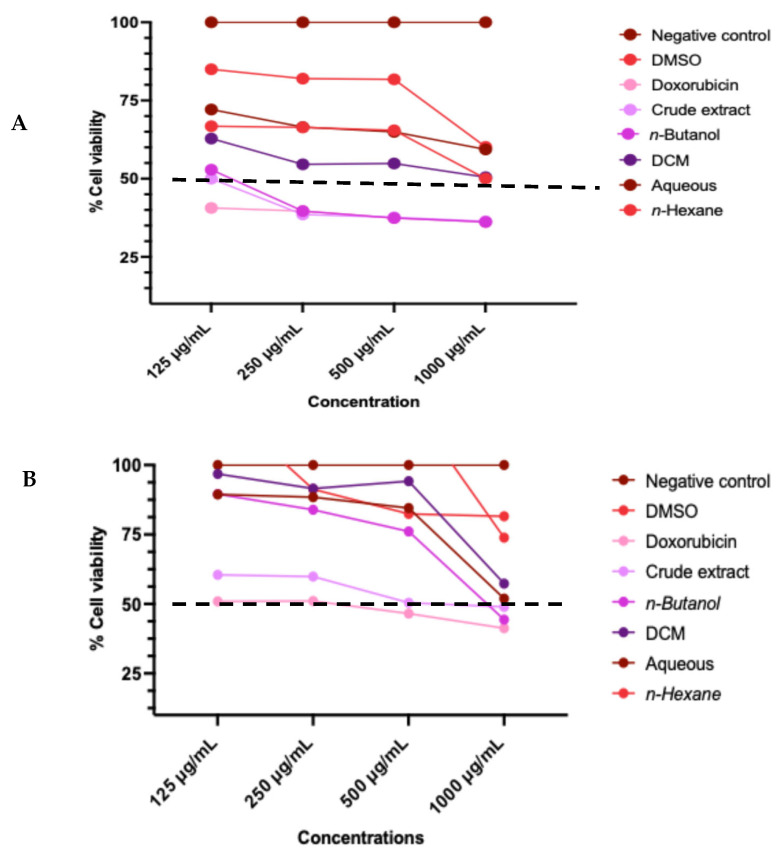

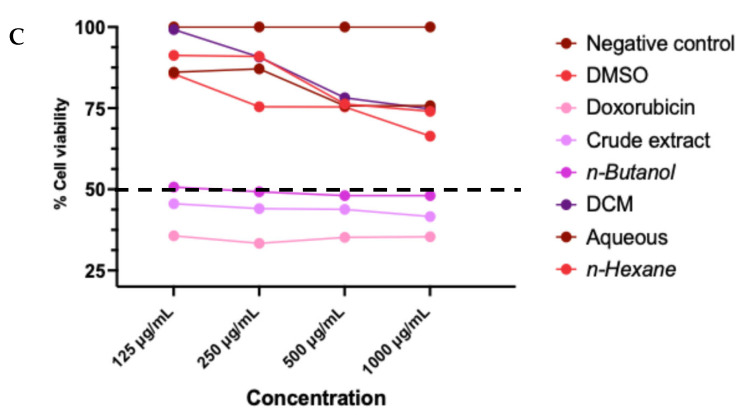
Cytotoxic effects of the crude extract and solvent fractions of *A. africanus* on MCF-7 (**A**), A549 (**B**) and HeLa (**C**) cells using the MTT assay. Cells were treated with varying concentrations (0.00–1000 µg/mL) of crude extract and fractions for 24 h. Cell viability was expressed as a percentage relative to the untreated control (*n* = 3; mean ± sd).

Cell viability assay was performed to evaluate the cytotoxic effects of the crude extract and solvent fractions of *A. africanus* (*n*-butanol, dichloromethane, *n*-hexane, and aqueous) on MCF-7, A549, and HeLa cancer cells at increasing concentrations (0.00–1000 µg/mL) (Figure 2). Untreated cells were used as a negative control, while doxorubicin (0.00–0.0034 µg/mL) was used as the positive control, and DMSO was used as a sample vehicle. The crude extract of *A. africanus* exhibited marked, concentration-dependent cytotoxic activity across all tested cell lines, with IC_50_ values of 130 µg/mL for MCF-7, 380 µg/mL for A549, and <125 µg/mL for HeLa cells, indicating greater sensitivity of the HeLa and MCF-7 cells compared to the A549 cell line. The *n*-Butanol fraction showed the strongest cytotoxic effect on MCF-7 cells (IC_50_ = 870 µg/mL). In contrast, the dichloromethane and *n*-hexane fractions displayed higher IC_50_ values on HeLa and A549 cell lines, indicating weaker cytotoxicity within the tested concentration ranges, with cell viability remaining above 73–87% across all tested concentrations (0.00–1000 µg/mL). The aqueous fraction had moderate activity across all three cell lines. A line was drawn on each toxicity graph to indicate a 50% viability in untreated control cells, to which all treatments were compared. Among the tested fractions, the *n*-butanol fraction and crude extract were selected and subsequently used for further assay due to their strong cytotoxic effects.

### 2.2. Morphological Profiling of Apoptosis Through DAPI Chromatin Staining, Annexin V/PI Membrane Labeling and Caspase-Glo^®^ 3/7 Luminescence

**Figure 3:**
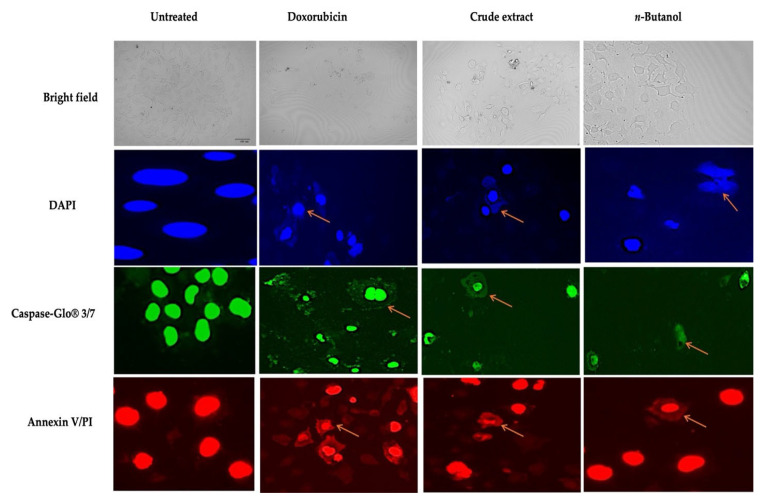
Morphology of MCF-7 cells treated with doxorubicin (positive control), crude extract, and the *n*-butanol fraction of *A. africanus* (125 µg/mL). Images were captured at ×40 magnification using randomly selected fields of view (≥5 fields per treatment). Cells were stained with DAPI (Blue) to visualise nuclear morphology, caspase 3/7 (green) to detect caspase activation, and annexin V/PI (red) micrographs to show morphological changes such as membrane damage, indicating untreated apoptosis as the negative control. Where the orange arrows show the morphological change.

DAPI staining was used to assess nuclear morphology (Figure 3) after treatment with crude and fractionated *A. africanus* (125 µg/mL). Doxorubicin was used as a positive control (0.0034 µg/mL). The nuclei appeared smaller, more condensed, and intensely stained in MCF-7 cells treated with doxorubicin and crude extract of *A. africanus*, compared to the untreated controls, which displayed uniformly round and intact nuclei with homogeneous fluorescence, indicating healthy, viable cells. These nuclear shrinkage and chromatin condensation are clear morphological hallmarks of apoptosis. Cells treated with an *n*-butanol fraction of *A. africanus* also showed signs of nuclear condensation, but to a lesser extent than those treated with crude extract and positive control doxorubicin. In parallel, the Caspase-Glo 3/7 assay was used to detect caspase activation, a biochemical indicator of apoptosis. MCF-7 Cells treated with doxorubicin, crude extract, and *n*-butanol fraction of *A. africanus* exhibited significant luminescence (*p* < 0.01), indicating robust activation of caspase 3/7, which confirms the high levels of apoptosis. Annexin V/PI micrographs demonstrated prominent bright red/orange, fluorescent nuclei in *A. africanus*-treated cells, consistent with apoptosis. Treatment with doxorubicin (positive control) displayed intense nuclear fluorescence, while crude extract and *n*-butanol fraction also induced apoptosis, but to a lesser extent than the positive control.

### 2.3. Quantitative Analysis of Apoptosis Using Caspase-Glo 3/7 Assay

**Figure 4:**
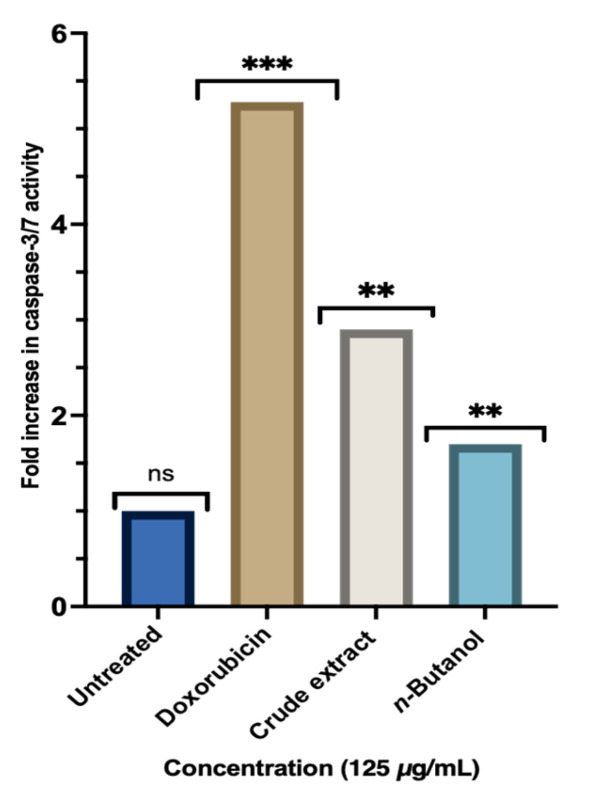
Qualitative analysis of apoptosis using the Caspase-Glo 3/7 assay. The graph shows capsase-3/7 activity in cells treated with crude extract and n-butanol fraction of *A. africanus*, with doxorubicin used as a positive control and untreated cells as a negative control. Data are presented as mean ± standard deviation. Statistical significance *p* < 0.001 (***) compared to control, (**) *p* < 0.001 compared to doxorubicin.

Quantification of Caspase-3/7 activity (Figure 4) showed that all treatments (125 µg/mL) produced measurable increases in apoptosis signalling compared with the untreated group. Doxorubicin produced the highest response, with a 5.28-fold increase relative to the untreated cells (*p* < 0.0001), consistent with its well-established role as a strong inducer of apoptosis. The crude extract of *A. africanus* also resulted in elevated caspase-3/7 activity, showing a 2.90-fold increase (*p* < 0.01), while the effect of *n*-butanol fraction was more moderate, corresponding to a 1.7-fold increase in activity (*p* < 0.01). Untreated cells maintained low baseline caspase activity (1.0-fold), confirming assay stability. Overall, although the extract produced variable degrees of caspase activation, the trend generally aligned with the Annexin V/PI data (below), indicating that apoptosis may occur.

### 2.4. Annexin V/PI Flow Cytometry Analysis of Apoptosis in Treated MCF-7 Cells

**Figure 5:**
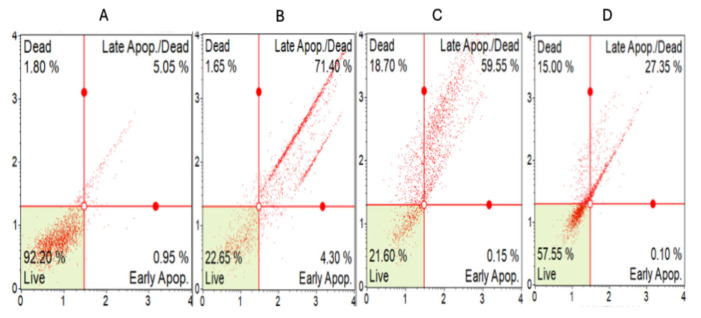

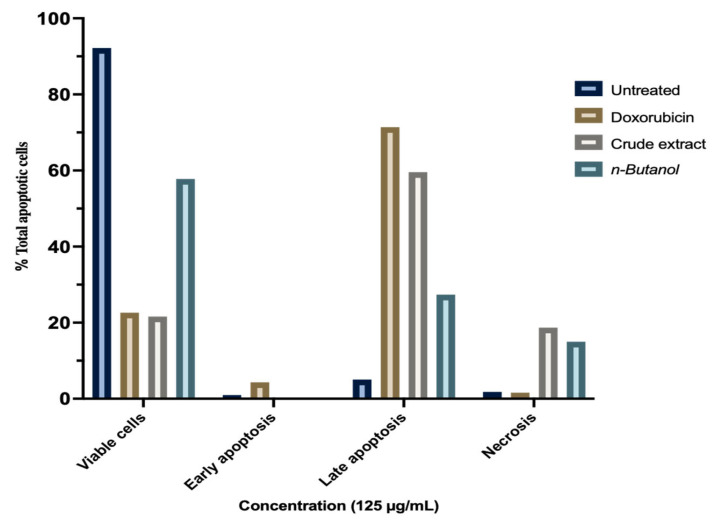
Apoptosis analysis of MCF-7 cells treated with crude extract, *n*-butanol fraction of *A. africanus* (125 µg/mL), compared with doxorubicin and negative control. Cells were stained with Annexin V/PI (red) to detect apoptotic and necrotic populations and analysed by flow cytometry to quantify different stages of cell death. Flow cytometry plots show the distribution of viable, early apoptotic, late apoptotic, and necrotic cells: (**A**) negative control; (**B**) doxorubicin (positive control); (**C**) crude extract; (**D**) *n*-butanol fraction. The bar graphs show the percentage cell population. Data are expressed as mean ± SD (*n* = 3). Significance of statistical differences (*p* < 0.05), additional intergroup comparisons were performed using Tukey’s post hoc test.

Induction of apoptosis was further confirmed using Annexin V/PI staining. Crude extract and *n*-butanol fraction (125 µg/mL) of *A. africanus* were processed for annexin V and PI staining according to the manufacturer’s instructions and then analysed by flow cytometry. The apoptotic cells stained with PI are indicated as a percentage of gated cells (Figure 5). The lower left quadrants of each panel represent the viable cells, which exclude PI and are negative for FITC-Annexin V binding. The upper left quadrants contain the non-viable, necrotic cells, which stained negative for FITC-Annexin V binding and PI uptake. The lower right quadrants represent early apoptotic cells, FITC-Annexin V positive and PI negative, and the upper right quadrants represent late apoptotic cells, FITC-Annexin V positive and PI positive. The apoptosis result presented in the Annexin V/PI graphs (Figure 5) showed a difference in apoptotic induction between the untreated control, doxorubicin, and *A. africanus*-treated samples. The untreated control showed minimal apoptotic activity, representing a baseline for comparison. Doxorubicin induced the highest level of apoptosis (75%), while treatment with the crude extract of *A. africanus* resulted in elevated apoptosis (60%) compared to the untreated control and the fractionated extract of *A. africanus*, which had a moderate apoptotic effect. 

### 2.5. Apoptotic Gene Regulation Induced by Agapanthus africanus

**Figure 6:**
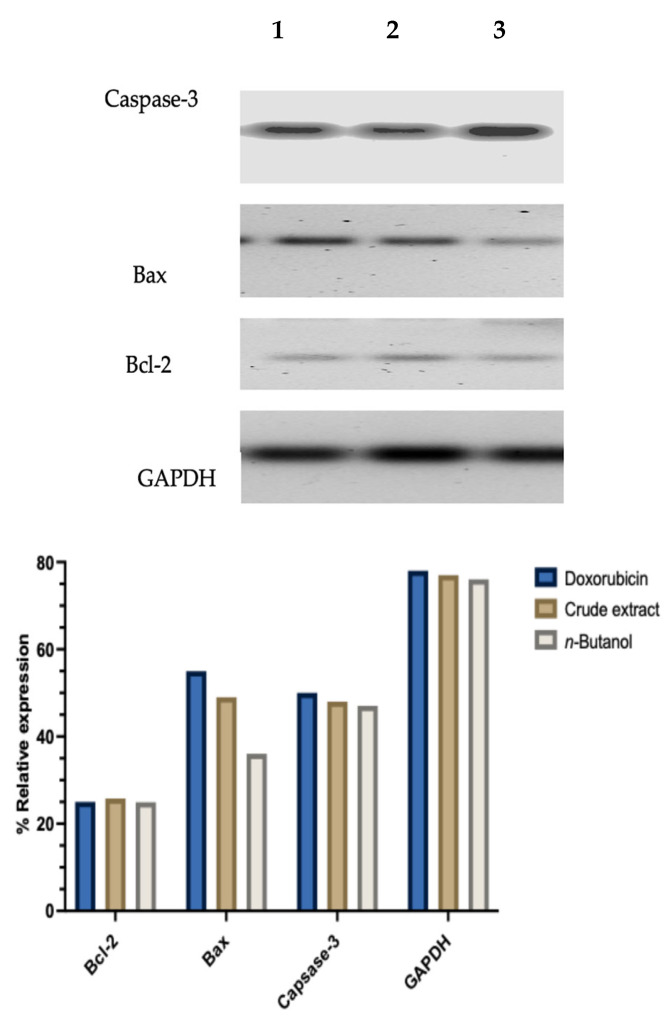
Gene expression of pro-apoptotic genes (*bax*, *caspase-3*), and anti-apoptotic gene (*bcl-2*) in MCF-7 cells treated with doxorubicin, *n*-butanol fraction of *A. africanus* and crude extracts of *A. africanus*. KEY: Lane 1, doxorubicin; Lane 2, crude extract; Lane 3, *n*-butanol fraction. The graph shows the relative band intensity (%) representing the expression levels of the apoptosis-associated genes.

Gel electrophoresis data, in Figure 6, showed clear differences in key apoptotic genes that responded to treatment with doxorubicin, crude and fractionated extract of *A. africanus*, treated on MCF-7 cells. Notably, there was a strong amplification of *caspase 3, bax*, and *GAPDH*, which illustrates high levels of gene expression. In contrast, the *bcl-2* bands appeared faint, suggesting that their expression was significantly lower. This pattern indicates that the plant extracts likely trigger apoptosis by increasing the expression of pro-apoptotic genes (*caspase-3* and *bax*) while suppressing the anti-apoptotic gene (*bcl-2*), supporting the involvement of the intrinsic apoptotic pathway. The consistency of expression of GAPDH, used as a housekeeping gene, confirmed the reliability of the results and the integrity of the RNA used (Figure 6).

### 2.6. Wound Healing Properties of Crude Extracts and n-Butanol Fraction of A. africanus on Mcf-7 Cells

**Figure 7:**
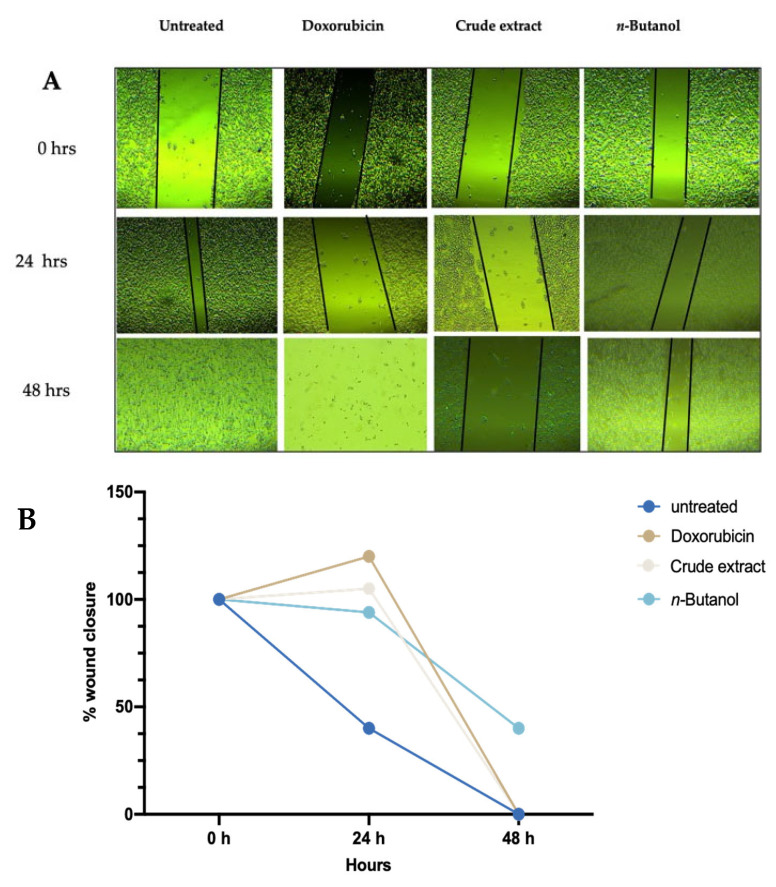
Representative images (**A**) from wound healing assay showing the impact of crude extract and *n*-butanol fraction on cell migration across the two-day incubation period, and the graph (**B**) illustrating the percentage of the remaining wound area following treatment with crude extract and *n*-butanol fraction. Doxorubicin was used as a positive control, and untreated cells served as a negative control.

The scratch wound-healing assay was used to evaluate the effects of the *A. africanus* crude extract and *n*-butanol fraction on cell migration. Representative images captured at 0 h, 24 h, and 48 h following treatment with doxorubicin, crude extract, and *n*-butanol are shown in Figure 7A. Doxorubicin served as the positive control, while untreated cells were used as the negative control. The corresponding quantitative analysis of wound area over the two days is presented in Figure 7B. Untreated cells demonstrated progressive wound closure, with the scratch area decreasing from 100% on 0 h to 40% after 24 h with complete closure after 48 h. In contrast, doxorubicin markedly impaired wound repair, resulting in an enlarged wound area (~131%) after 24 h and the absence of a measurable monolayer after 48 h. Cells treated with the crude extract also exhibited impaired migration as indicated by a slight increase in wound size after 24 h (~103–105%) and further widening after 48 h (~120%). The *n*-butanol fraction produced a less pronounced wound healing effect, with partial closure after 48 h (~40–42%).

### 2.7. Phytochemical Profiling Using GC-MS Analysis

**Figure 8:**
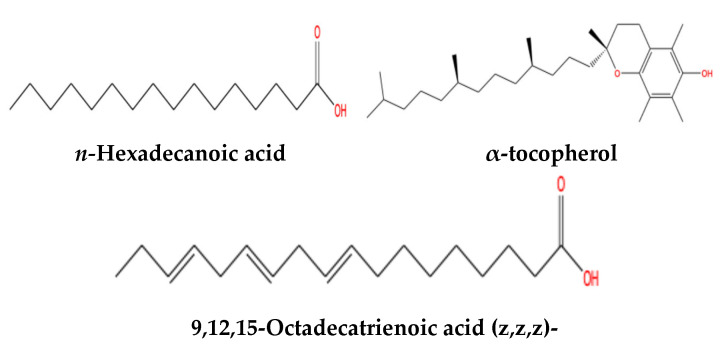
Chemical structures of the most dominant volatile compounds found in the *n*-butanol fraction of *A. africanus* through GC-MS.

**Figure 9:**
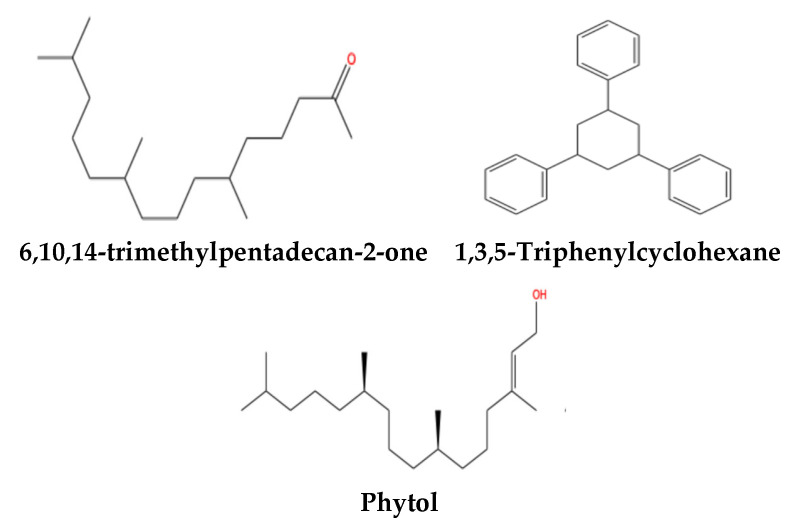
Chemical structures of the most dominant volatile compounds found in the crude extract of *A. africanus* through GC-MS.

GC-MS analysis was used to identify and quantify different metabolites of the *n*-butanol fraction and crude extract of *A. africanus* (Table 1). In the present study, 24 volatile compounds were identified and eluted between 2.12 and 33.652 min. Properties of the detected volatile compounds, including retention time, molecular weight, peak area %, and compound classifications, are outlined in Table 1. Identified compounds included *n*-hexadecanoic acid; 9,12,15-octadecatrienoic acid; 4-hydroxy-4-methyl-2-pentanone; 5-methyl-2-phenyl *α*-Tocospiro A; vitamin E, and phytyl decanoate. Major constituents for *n*-butanol fraction (Figure 8) were 9,12,15-octadecatrienoic acid (2.02%), *n*-hexadecanoic acid (1.54%) and vitamin E (0.87%) with retention times of 22.52, 20.81 and 33.31 min, respectively. Minor compounds included 4-hydroxy-4-methyl-2-pentanone (0.65%), dodecanoic acid (0.21%), and phytyl derivatives (0.86%). The GC–MS profile of the crude extract was dominated by phytol (6.48%) as the most abundant constituent (Figure 9). Other prominent compounds included 6,10,14-trimethyl 2-pentadecanone (2.31%), a branched aliphatic ketone, and 9,12,15-octadecatrienoic acid (2.02%), a polyunsaturated fatty acid. In addition, *n*-hexadecanoic acid (palmitic acid) (1.54%) and 1,3,5-triphenylcyclohexane (1.28%) were present in appreciable amounts. Collectively, these compounds represent the dominant chemical constituents of the crude extract and are likely to contribute substantially to its biological activity (Table 2).

Compounds were putatively identified based on GC–MS spectral matching with the NIST library and comparison of retention times reported in the literature. Peak areas represent relative abundance normalised to the total ion chromatogram (TIC).

This study identified 23 volatile compounds, but only the chemical structures of the most dominant volatile compounds within the *A. africanus* are shown in Figure 8 and Figure 9.

**Figure 10:**
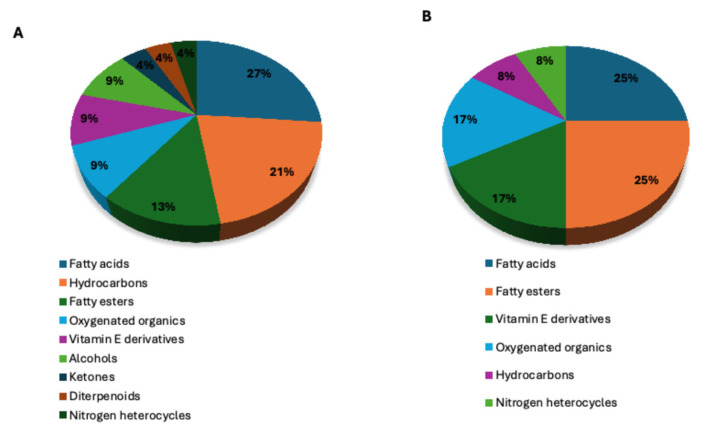
GC–MS-based distribution of compound classes in *Agapanthus africanus*, (**A**) the crude extract and (**B**) the *n*-butanol fraction.

Figure 10A shows the distribution classes identified in *A. africanus.* The *n*-butanol fraction (Figure 10A) was dominated by fatty esters and fatty esters (25%) each, followed by oxygenated organic compounds and vitamin E derivatives (17% each). Hydrocarbons and nitrogen-containing heterocycles were present in lower proportions (8.3% each). In contrast, the crude extract (in Figure 10B) showed more diverse chemical profiles, with fatty acids (26%) and hydrocarbons (22%) as the major classes. Followed by fatty esters (13%). Vitamin E derivatives, alcohols, and other oxygenated organic compounds each contributed 8.7%, while ketones, diterpenoids, and nitrogen-containing heterocycles were minor constituents (4.3% each).

## 3. Discussion

*A. africanus* has a history of use in traditional medicine for various ailments, yet its scientific validation, particularly concerning apoptotic activity, remains largely unexplored. The present study investigated the chemical composition, cytotoxicity, pro-apoptotic effects, and the underlying mechanism of action of the crude extract, aqueous, *n*-hexane, DCM, and *n*-butanol fractions of leaf extracts of *A. africanus* on MCF-7, A549, and HeLa cell lines in vitro. Cell viability potential of crude extract and fractionated *A. africanus* was investigated against MCF-7, A549, and HeLa cells using the MTT assay. *n*-Butanol and crude extract demonstrated the strongest cytotoxic activity in all the cell lines (Figure 2A–C), specifically against MCF-7 cells with an IC_50_ value of 870 and 130 µg/mL, respectively, and HeLa cells with an IC_50_ value of 1.41 mg/mL and 125 µg/mL. This suggests a degree of cell line selectivity, which is desirable in anticancer drug discovery as it indicates potential for targeted action. In contrast, the DCM and *n*-hexane fractions exhibited weaker cytotoxicity, with the highest IC_50_ values recorded for A549 cells. Interestingly, the aqueous fraction also showed moderate cytotoxicity across the cell lines, with the lowest IC_50_ value against A549 cells. The superior activity of the *n*-butanol fraction over the DCM and *n*-hexane fractions highlights the importance of solvent polarity in concentrating bioactive constituents [16]. The crude extract exhibited higher cytotoxic activity than the *n*-butanol fraction, as indicated by its lower IC_50_ value. This enhanced activity correlates with the greater chemical diversity of the crude extract, which contained multiple bioactive classes, including diterpenoid alcohols, fatty acids, ketones, hydrocarbons, and vitamin E derivatives. Phytol, the dominant constituent, has been reported to induce cytotoxic and pro-apoptotic effects in cancer cell lines [27]. The broader compound profile of the crude extract likely promoted additive or synergistic effects, whereas fractionation reduced this complexity, resulting in lower cytotoxic activity of the n-butanol fraction.

The annexin V-binding assay is the most sensitive and widely used technique to detect and differentiate between late apoptosis and early apoptosis, as well as between apoptotic and necrotic cells [29]. In this present study, the treatment with the crude extract of *A. africanus* resulted in elevated total apoptosis of about 60% compared to the fractionated extract of *A. africanus*, which showed approximately 27% (Figure 5). This indicates reduced presence of key bioactive compounds in fractionated extracts of *A. africanus*. This finding aligns with previous phytochemical studies that reported that *A. africanus* contains secondary metabolites such as saponins, flavonoids, and phenolic compounds, which are often associated with anticancer properties [30]. For instance, Gomaa et al. [19] demonstrated that extracts of *A. africanus* possess potent antiproliferative activity and antioxidant activity, which may mediate apoptosis through ROS modulation. The observed apoptotic effects might also be attributed to the synergistic action of multiple bioactive compounds within the crude extracts, which are more efficacious in combination than in isolation due to synergistic mechanisms, affecting multiple targets simultaneously [31,32].

Apoptosis is a desirable outcome in cancer treatment, and its induction is a key marker of the therapeutic potential of plant-derived compounds [33]. In this study, the ability of *A. africanus* crude extract and fractions to induce apoptosis was evaluated using DAPI nuclear staining and the Caspase-Glo 3/7 assay. DAPI staining is a fluorescence-based method that highlights the structural changes in the nucleus of apoptotic cells [34]. Under fluorescence microscopy, healthy cell nuclei appear uniform, round, and lightly stained, while the apoptotic nuclei show bright, fragmented chromatin due to DNA compaction and cleavage. In this study, both crude and fraction of *A. africanus* caused visible nuclear fragmentation, suggesting that the compounds present in *A. africanus* may trigger DNA damage. These morphological changes are typical of the early to late stages of apoptosis and are in line with the results observed in annexin V/PI which showed elevated apoptosis in the crude extract. To strengthen the evidence, the Caspase-Glo 3/7 assay assessed caspase enzyme activity, specifically caspase-3 and caspase-7, which are critical executors of apoptosis [35]. In this study, an increased luminescence was observed in both the crude and the fraction, confirming activation of the execution phase of apoptosis. Crude extract of *A. africanus* and its *n*-butanol fraction produced measurable apoptosis responses across the caspase-3/7 performed, although the extent of these effects varies. The increases in caspase-3/7 activity suggest some involvement of executioner caspases, which play a central role in the final stages of apoptosis [17,36]. However, these responses were lower than those produced by doxorubicin. The crude extract generally produced a stronger response than the *n*-butanol fraction, which may be attributed to the broader range of phytochemicals present in an unfractionated extract. Synergistic interactions between plant constituents are well documented and often contribute to greater biological activity compared with isolated fractions [37].

The expression levels of *caspase-3*, *bax*, and *bcl-2* were evaluated using gel electrophoresis (Figure 6). While the expression level of glyceraldehyde 3-phosphate dehydrogenase (GAPDH) mRNA remained consistent across all treatment groups in MCF-7 cells, indicating that an equal amount of RNA was used and successfully amplified in each sample [38]. The results revealed the increased expression of *bax* and *caspase-3*, which are both pro-apoptotic markers, strongly suggesting that *A. africanus* extracts are promoting cell death through the intrinsic mitochondrial pathway. *Bax* plays a central role by destabilising the mitochondrial membrane, which leads to the release of cytochrome c, a signal that activates Caspase-9, which is one of the key initiator caspases [18,39]. Caspase-9 then triggers a cascade involving executioner caspases like Caspase-3 and Caspase-7, ultimately driving the cell into apoptosis. Moreover, the downregulation of *bcl-2* by *A. africanus*, which is an anti-apoptotic protein in the present study, further reinforces the shift toward programmed cell death. This is consistent with the observed mitochondrial-dependent caspase activation and supports the involvement of the *bax/bcl-2* ratio as a key regulator of mitochondrial outer membrane permeabilisation [17,40]. Although the magnitude of gene modulation varied among treatments, the overall pattern confirmed that both the crude extract and *n*-butanol fraction of *A. africanus* trigger mitochondrial-mediated apoptosis, consistent with the flow cytometry and caspase activation data. These findings are consistent with previous studies where steroidal glycosides isolated from *A. africanus* roots induced apoptosis in small-cell lung cancer cells [14]. Similarly, *Agapanthussaponin A* was reported to trigger both apoptosis and ferroptosis via caspase-3/9 activation [20], supporting the pro-apoptotic potential observed in the present study.

The study also evaluated cell migration using the scratch assay. The crude extract of *A. africanus* appeared to limit the closure of the scratch, as the wound area increased after 24 h and widened further after 48 h. This pattern suggests a possible reduction in cell viability in the presence of the crude extract. In comparison, the *n*-butanol fraction showed only a slight decrease in wound size after 24 h of treatment and partial closure after 48 h, indicating that the fraction may have exerted a weaker inhibitory effect on cell migration. These trends align with previous observations that the wound-healing response can be influenced by the cytotoxic characteristics and composition of plant-derived extracts [41].

Chemical constituents responsible for the observed apoptotic effects of *A. africanus* leaf extracts were identified using a *n*-butanol fraction using GC-MS analysis. Due to the nature of GC-MS, which fragments bioactive compounds during the heating process, the representative phytoconstituents were determined by analysing the mass spectral data from the integrated chromatogram peaks of the plant (Figure 8). The GC-MS analysis of the *n*-butanol fraction of *A. africanus* showed 16 major compounds, while the crude extract of *A. africanus* showed 23 compounds, of which some of the compounds identified have been associated with anticancer properties (Table 1 and Table 2).

The identified compounds included fatty acids, esters, ketones, vitamin E, alcohols, and hydrocarbon derivatives, many of which have been previously reported to possess anticancer properties. For example, recent studies have shown the cytotoxic potential of phytochemicals such as *n*-hexadecanoic acid against different cancer cell lines [24]. The *n*-butanol extract induced apoptosis and autophagy, evidenced by increased expression of *caspase-3, caspase-8*, and *bax*, as well as the conversion of LC3-l to LC3-ll and upregulation of beclin-1 [42]. Moreover, *n*-hexadecanoic acid has shown selective cytotoxicity against human leukemic cells, and the mechanisms involved the inhibition of DNA topoisomerase I and induction of apoptosis [43].

Another predominant compound with medical benefits isolated from *A. africanus* was 9,12,15-octadecatrienoic Acid (ALA), known as α-linolenic acid. Fan et al. [44] showed that ALA can suppress the proliferation of different cancer cells, such as osteosarcoma MG63 cells, by a mechanism that involves the inhibition of fatty acid synthase (FASN), a key enzyme in lipid biosynthesis, which is often upregulated in cancer cells. In other analyses, ALA has been identified as the major constituent in various plant extracts exhibiting anticancer activities. For example, the methanolic extract of *Tephrosia perpuea* subsp. *Apollinea* contained significant amounts of ALA, contributing to its observed anticancer effects on multiple cancer cell lines [45]. Previous studies have also shown that vitamin E (γ-tocotrienol) significantly activates ERK signaling, which is essential for its pro-apoptotic effects [46]. While the detection of some volatile compounds was reported in previous studies, the presence of 4-hydroxy-4-methyl-2-pentanone, 3-benzyloxy-1,2-diacetyl-1,2-propanediol, 5-methyl-2-phenyl-1H-indole, and 5-methyl-2-phenyllindolizine is reported for the first time from *A. africanus*. Additionally, *n*-hexadecanoic acid and dodecanoic acid, both identified in the GC-MS profiles of the *n*-butanol fraction of *A. africanus*, have demonstrated pro-apoptotic effects in various cancer cells by inducing mitochondrial dysfunction and ROS-mediated stress [47]. Together, these compounds likely work synergistically to drive the observed apoptotic responses, supporting the idea that *A. africanus* could offer multi-targeted anticancer potential through intrinsic cell death mechanisms. GC–MS analysis is limited to volatile and semi-volatile compounds; thus, polar constituents in the *n*-butanol fraction and crude extract may not have been detected and could also contribute to the observed cytotoxic and apoptotic effects. Previous phytochemical studies on *Agapanthus africanus* have predominantly focused on the underground parts of the plant, particularly the rhizomes and roots, which are reported to be rich in steroidal saponins [30]. In contrast, the present study investigated leaf extracts and revealed a distinct phytochemical profile characterised by non-saponin constituents, including phenolic compounds and fatty acid derivatives. This clear compositional difference highlights organ-specific secondary metabolite distribution within *A. africanus* and emphasises the importance of plant part selection when evaluating biological activity.

## 4. Methods and Materials

### 4.1. Plant Collection and Extraction

Fresh leaves of *Agapanthus africanus* were collected in September 2021 from the South African National Biodiversity Institute (SANBI) in Nelspruit, Mpumalanga Province, from a cultivated population. The leaves were authenticated by a botanist (Mrs Windfred Nqwenya), and voucher specimens were lodged in the Ward herbarium at SANBI for future reference. The leaves were washed, air-dried at room temperature (25 °C) for 7 days, and ground into a fine powder. Using a PX-MFC90 Poly-Mix laboratory mill, Kinematic AG, Phoenix, AZ, USA. For extraction, 500 g of powdered leaves were soaked in 1 L of acetone at room temperature (25 °C) for 48 h with occasional stirring (solid-to-solvent ratio of 1:2 *w*/*v*). The mixture was filtered using Whatman No. 1 filter paper, and the filtrate was concentrated under the fume hood to yield the crude acetone extract, following the method described by Isa et al. [48].

### 4.2. Liquid–Liquid Fractions

Using a sequential solvent extraction method in a separation funnel, the crude plant extract was liquid–liquid partitioned [48]. The crude extract was dissolved in 120 mL of distilled water and ethanol (3:1) and transferred to a separating funnel. An equal volume of n-hexane was also added to the funnel, shaken vigorously, and left to separate. The aqueous layer was kept, while the *n*-hexane layer was collected. This step was repeated three times. The same process was followed by dichloromethane (DCM) and *n*-butanol solvents. The final remaining aqueous layer was retained as the residual aqueous fraction. This method allowed for the separation of plant compounds based on polarity. Each fraction was concentrated under the fume hood and weighed to determine yield. The yields were as follows: n-hexane (11.4 g), DCM (5.6 g), n-butanol (1.7 g), and aqueous (10.3 g). All extracts were filtered and stored at 4 °C until further use.

### 4.3. Cell Culture and Cytotoxicity Assay

MCF-7, A549, and HeLa cells were obtained from Sigma-Aldrich in South Africa and cultured separately in T75 flasks (Sigma-Aldrich, St. Louis, MO, USA). Cancer cells were cultured in Dulbecco’s Modified Eagle Medium (DMEM) (Sigma-Aldrich, USA) supplemented with 10% fetal bovine serum (FBS) and maintained at 37 °C with 5% CO_2_. Cytotoxicity was assessed using a 3-(4,5-dimethylthiazol-2-yl)-2,5-diphenyltetrazolium bromide (MTT) assay, following the method described [49]. MCF-7, A549, and HeLa cells were maintained at 70–80% confluency before subculture. Cells were seeded in 96-well plates (1 × 10^6^ cells per well) and treated with different concentrations (0.00–1000 µg/mL) of the extracts for 24 h. After incubation, 20 μL of MTT reagent (5 mg/mL in PBS) was added to each well and incubated for 4 h at 37 °C. Formazan crystals formed were dissolved in 100 μL of DMSO, and absorbance was measured at 570 nm using the Glomax Multi + Detection luminometer System (Promega, Madison, WI, USA) [50]:

(1) $$\mathrm{Viability}\;(\%)\;=\;\frac{Absorbance\;of\;the\;treated\;sample}{Absorbance\;of\;the\;control}\times 100$$

### 4.4. Apoptosis Detection (Annexin V/PI) Flow Cytometry

Apoptosis was assessed using the Annexin V/PI reagents and analysed on Guava^®^ Muse^®^ Cell Analyser (Fremont, CA, USA) according to the manufacturer’s protocol. Briefly, cells (1 × 10^6^ cells/mL) were harvested following treatment, washed twice with PBS, and resuspended in 100 µL of the assay buffer. Then, 100 µL of the Annexin V/PI reagent was added, and the samples were incubated in the dark at room temperature for 20 min. Samples were analysed on Guava^®^ Muse^®^ Cell Analyser, which uses microcapillary-based flow cytometry to distinguish between viable cells, early apoptotic, late apoptotic, and necrotic cells. Data were acquired and analysed using the Muse^®^ Analyser Software (cat. no. 0500-3115; Luminex) and further analyzed using Muse analysis software (version 1.5; Luminex).

### 4.5. DAPI Nuclear Staining and Caspase-Glo^®^ 3/7 Assay

To assess morphological changes associated with apoptosis, 4′,6-diamidino-2-phenylindole (DAPI) and caspase-Glo^®^ 3/7 staining were performed following the method described by Daniel and DeCoster [51,52]. Treated and control cells were seeded (1 × 10^6^ cells per well) in 24-well plates and incubated with the respective extracts for 24 h. Cells were then fixed in 4% paraformaldehyde for 15 min at room temperature, washed with phosphate-buffered saline (PBS), and stained with DAPI (1 µg/mL) for 10 min in the dark. and for caspase-Glo^®^ 3/7 activity, caspase-Glo^®^ 3/7 assay system (Promega (Madison, WI, USA), catalog number: G8090) was used, an equal volume of Caspase-Glo^®^ 3/7 reagent was added to each well and mixed gently. The plate was incubated at room temperature in the dark for 30 min, allowing the caspase enzyme to cleave the luminogenic substrate, generating a luminescent signal proportional to caspase-3/7 activity. Nuclear morphology was examined under a ZOE^TM^ Fluorescent Cell Imager (BioRad, Hercules, CA, USA).

### 4.6. Quantification Analysis of Apoptosis Using Caspase-Glo^®^ 3/7 Assay

Caspase-Glo 3/7 enzymatic activity was measured using the Caspase-Glo^®^ 3/7 luminescent assay according to the manufacturer’s instructions. Briefly, cells were seeded into a 96-well plate (1 × 10^6^ cells/mL) and allowed to attach overnight. Cells were treated with *A. africanus* extracts for 24 h. Before the assay, both the plate and Caspase-Glo^®^ 3/7 reagent were equilibrated to room temperature for 30 min. caspase-Glo^®^ 3/7 reagent was then added directly to each well at a 1:1 ratio (100 µL reagent to 100 µL sample), followed by gentle mixing for 30 min. Plates were incubated for 45 min at room temperature in the dark to allow cell lysis and cleavage of the luminogenic substrate. Luminescence, proportional to caspase-3/7 activity, was measured using a microplate reader, Promega Glomax-Multi-Detection System. Caspase activity was expressed as relative luminescence units (RLU) and normalised to the untreated control [53].

### 4.7. Gene Expression of the Plant Extracts

Gene expression analysis was performed using reverse transcription polymerase chain reaction (RT-PCR). Total RNA was extracted from treated and control MCF-7 breast cancer cells using the Omega E.Z.N.A HP total RNA isolation kit (BIO-TEK, Winooski, VT, USA), following the manufacturer’s protocol. The quality and concentration of RNA were assessed using an IMPLEN GMBH Nanophotometer N50 Touch (United Scientific, Libertyville, IL, USA, serial number: 151404), made in Germany. Equal amounts of RNA were then reverse transcribed into cDNA using Luna Script RT superMix kit (BIORAD) following the manufacturer’s protocol. PCR amplification was conducted for specific apoptotic genes *caspase-3* (forward: 5′-CCATGGGTAGCAGCCTCCCTTC-3′; reverse: 5′-TGCGCTGCTCTGCCTTCT-3′); *bax* (forward: 5′-TCCCCCCAGAGGTCTTTT-3′; reverse: 5′-CGGCCCAGTTGTTGAAGTTG-3′), (*bcl-2*; forward: 5′-CTGCACCTGACGCCCTTCACC-3′); reverse: 5′-CACATGACCCCACCGAACTCAAAGA-3′); and GAPDH (forward: 5′-TGCGCTGCTGCTCTGCC-3′; reverse: 5′-CCATGGGTAGCAGCTCCTTC-3′) as a housekeeping gene for normalisation. The reaction began with an initial denaturation step at 95 °C for 3 min. This was followed by 32 cycles consisting of denaturation at 95 °C for 30 s, annealing at a temperature ranging between 50.32 °C and 65.55 °C, and extension at 72 °C for 45 s. A final extension was performed at 72 °C for 7 min. Amplified products were visualised using agarose gel electrophoresis using the CHEMDOC instrument, and band intensity was used to compare gene expression [38,54].

### 4.8. Wound Healing Properties of Crude Extracts and n-Butanol Fraction of A. africanus on Mcf-7 Cells

The wound closure capability of the MCF-7 cells was assessed using a scratch assay with slight modification [41]. The cells were cultivated in RPMI medium (supplemented with 5% L-glutamine, 10% FBS, and 5% pen-strep) for 24 h to reach 70–80% confluency. A vertical artificial wound was created using a 10 µL pipette tip. The cells were subsequently rinsed to eliminate the floating cells. The cells were treated with crude extract and n-butanol fraction of *A. africanus* at a concentration of 1000 µg/mL for 72 h. Microscopic images of the same area of the respective scratch were taken using a Nikon Eclipse TS100 at a magnification of 10×. The open wound area was determined by measuring the distance between the lines using a ruler. Wound closure in percentage was calculated by the following method described by Balko et al. [55]. Wound area was estimated using the formula:

(2) $$\mathrm{Wound}\;\mathrm{area}\;(\%)\;=\;\frac{open\;wound\;area\;at\;specified\;time}{open\;wound\;area\;0\;h}\times 100$$

### 4.9. Phytochemical Profiling Using GC-MS Analysis

Gas Chromatography–Mass Spectrometry (GC-MS) analysis was employed to identify major phytochemical constituents present in the N-butanol fraction of *A. africanus*, using ChemStation Integrator-autoInt system (Agilent Technologies, Santa Clara, CA, USA) at Protechnik Laboratories (ARMSCOR SOC Ltd., Pretoria, South Africa). Sample preparation and analysis were conducted following the protocol by [46]; subsequently, the sample was re-dissolved in HPLC-GRADE methanol. A sample (1 µL) was injected in splitless mode into a GC-MS system equipped with a HP-5MS capillary column (30 m × 0.25 mm internal diameter, 0.25 µm film thickness). The carrier gas is helium (99.99% purity) at a constant flow rate of 1.0 mL/min. The oven temperature was programmed as follows: an initial temperature of 50 °C (held for 3 min), increased at 10 °C/min to 280 °C, and held for 10 min. The injector and mass transfer line temperatures were maintained at 250 °C and 280 °C, respectively. The mass spectrometer was operated in electron ionisation mode at 70 eV, with a mass scan range of 50–550 *m*/*z*. Chromatographic peaks were automatically integrated and processed using ChemStation software version E.02.02.1431. The mass spectra of the detected compounds were interpreted and compared with reference spectra from the National Institute of Standards and Technology (NIST 2017) spectral library to confirm compound identities.

### 4.10. Statistical Analysis

All experiments were performed in triplicate, and the data are expressed as mean ± standard deviation (SD). Statistical analysis was carried out using Microsoft Excel, applying one-way analysis of variance (ANOVA) to determine significant differences between treatment groups. A *p*-value ≤ 0.05 was considered statistically significant.

## 5. Conclusions

In conclusion, the cell viability assay demonstrated that treatment with crude extract and different fractions of *A. africanus* led to a dose-dependent reduction in cell viability across the tested human cancer cell lines. Among the treatments, n-butanol fraction, together with crude extract, exhibited greater cytotoxic effects than the DCM, aqueous and n-hexane fractions against MCF-7, A549, and HeLa cells, with IC_50_ values of 130 µg/mL, 380 µg/mL, and <125 µg/mL, respectively. In contrast, *n*-hexane, dichloromethane and the aqueous fractions exhibited higher IC_50_ values against cancer cells, suggesting the most active compounds may be semi-polar to non-polar in nature. DAPI nuclear staining revealed morphological features consistent with apoptosis, such as chromatin condensation and nuclear fragmentation. Additionally, the Caspase-glo 3/7 assay provided additional mechanistic support, indicating an activation of caspases (3 and 7) in the treated cancer cells. The crude extract increased caspase activity by 2.9-fold, while the *n*-butanol fraction induced a 1.7-fold rise compared with untreated cells. Quantitative flow-cytometric analysis confirmed a marked increase in late-apoptotic cell population following treatment with both the crude extract (~60%) and n-butanol fraction (~27%) of *A. africanus*, consistent with observations from DAPI nuclear staining and Caspase-Glo 3/7 activation. Together, these results suggest *A. africanus* triggers programmed cell death rather than necrosis. The gene expression of apoptosis-related genes revealed an upregulation of pro-apoptotic genes (*bax* and *Caspase-3*) and downregulation of the anti-apoptotic gene (*bcl-2*). The stable expression of GAPDH verified the reliability of the normalisation process. Complementary GC-MS profiling of the most active fraction identified the presence of bioactive phytoconstituents such as *n*-hexadecanoic acid, α-tocopherol, and octadecanoic acid compounds known for their cytotoxic, antioxidant, and pro-apoptotic properties. The chemical diversity detected supports the biological data and provides a molecular basis for the anticancer potential of *A. agapanthus*. Although the present results confirm caspase-3/7 activation, nuclear fragmentation, and *bax/bcl-2* modulation, additional mechanistic studies are recommended to determine the upstream signalling pathways involved. Techniques such as Western blotting could validate apoptosis at the protein level.

## Figures and Tables

**Table 1:** Volatile compounds of the *n*-butanol fraction and crude extract of *A. africanus*.

Compound Name	Molecular Formula	MW (g/mol)	RT (min)	Peak Area (%)	Nature of Compound	*n*-Butanol Fraction	Crude Extract
4-Hydroxy-4-methyl-2-pentanone	C_6_H_12_O_2_	116.16	5.832	0.65	ketone	✓	✓
Dodecanoic acid	C_12_H_24_O_2_	200.32	16.469	0.21	fatty acid	✓	✓
Tetradecanoic acid	C_14_H_28_O_2_	228.37	18.72	0.21	fatty acid	—	✓
Pentadecanoic acid	C_15_H_30_O_2_	242.40	18.27	0.57	fatty acid	—	✓
*n*-Hexadecanoic acid	C_16_H_32_O_2_	256.43	20.809	1.54	fatty acid	✓	✓
9,12,15-Octadecatrienoic acid	C_18_H_30_O_2_	278.43	22.509	2.02	fatty acid	✓	✓
Cyclohexane	C_6_H_12_	84.16	25.337	0.30	Hydrocarbon	✓	—
1,3,5-Triphenylcyclohexane	C_24_H_24_	312.46	25.340	1.28	Hydrocarbon	✗	✓
3-Benzyloxy-1,2-diacetyl-1,2-propanediol	C_14_H_18_O_5_	266.29	25.337	0.30	organic	✓	✓
5-methyl-2-phenyl-1*H*-indole	C_15_H_13_N	207.28	26.243	0.64	Nitrogen-containing heterocycle	✓	✓
6,7-Dimethyl-1,2,3,5,8,8a-hexahydronaphthalene	C_12_H_18_	162.27	12.07	0.68	hydrocarbon	—	✓
1-Docosene	C_22_H_44_	308.60	19.10	0.70	alkene	—	✓
Neophytadiene	C_20_H_38_	278.52	19.61	0.50	hydrocarbon	—	✓
6,10,14-trimethylpentadecan-2-one	C_18_H_36_O	268.48	19.68	2.31	Branched aliphatic ketone	—	✓
1-Tetracosene	C_24_H_48_	336.64	21.13	0.87	Long-chain alkene	—	✓
1-Heptatriacotanol	C_37_H_76_O	537.01	21.85	0.78	Long-chain fatty alcohol	—	✓
Nonadecane	C_19_H_40_	268.52	21.75	0.57	Long-chain aliphatic hydrocarbon	—	✓
Phytol	C_20_H_40_O	296.54	22.30	6.48	Diterpenoid alcohol	—	✓
α-Tocospiro A	C_29_H_50_O_4_	462.72	29.233	0.49	Vitamin E derivative	✓	✓
Tricosanoic acid	C_23_H_46_O_2_	354.57	29.25	0.58	Very-long-chain saturated fatty acid	—	✓
α-tocopherol	C_29_H_50_O_2_	430.71	33.314	0.87	Vitamin E	✓	✓
Phytyl dodecanoate	C_32_H_62_O_2_	478.80	33.652	0.86	Fatty acid ester	✓	✓
Phytyl 2-methylbutanoate	C_25_H_48_O_2_	380.60	33.652	0.86	Fatty acid ester	✓	✓
Phytyl decanoate	C_30_H_58_O_2_	450.80	33.652	0.86	Fatty acid ester	✓	✓

**Table 2:** Biological activities of volatile compounds found in the *n*-Butanol fraction and crude extract of *A. africanus* through GC-MS.

Name of Compound	Biological Activity	References
Dodecanoic acid	Anticancer, anti-inflammatory	[21,22]
*n*-Hexadecanoic acid	Anticancer, antioxidant	[23,24]
9,12,15-Octadecatrienoic acid	Anticancer, antioxidant, and anti-inflammatory	[24,25]
α-Tocospiro A	Anticancer	[26]
Phytol	Anticancer, antioxidant	[24,27,28]
Saturated fatty acids (e.g., Tetradecanoic acid)	Anticancer, anti-inflammatory	[21,22]

## Data Availability

The original contributions presented in this study are included in the article. Further inquiries can be directed to the corresponding author.
